# Forecasting Ecological Genomics: High-Tech Animal Instrumentation Meets High-Throughput Sequencing

**DOI:** 10.1371/journal.pbio.1002350

**Published:** 2016-01-08

**Authors:** Aaron B. A. Shafer, Joseph M. Northrup, Martin Wikelski, George Wittemyer, Jochen B. W. Wolf

**Affiliations:** 1 Uppsala University, Department of Ecology and Genetics, Uppsala, Sweden; 2 Colorado State University, Department of Fish, Wildlife, and Conservation Biology, Fort Collins, Colorado, United States of America; 3 Max Planck Institute for Ornithology, Radolfzell, Germany; 4 University of Konstanz, Biology, Konstanz, Germany

## Abstract

Recent advancements in animal tracking technology and high-throughput sequencing are rapidly changing the questions and scope of research in the biological sciences. The integration of genomic data with high-tech animal instrumentation comes as a natural progression of traditional work in ecological genetics, and we provide a framework for linking the separate data streams from these technologies. Such a merger will elucidate the genetic basis of adaptive behaviors like migration and hibernation and advance our understanding of fundamental ecological and evolutionary processes such as pathogen transmission, population responses to environmental change, and communication in natural populations.

## Abstract 2

This essay explores the potential synergy between recent technological advances in animal tracking and DNA sequencing, which puts research in the biological sciences on the brink of a revolution.

## Introduction

Biological research is guided by a series of unifying concepts, ranging from Darwin’s theory of evolution and the modern synthesis to optimal foraging and Hubbell’s unified neutral theory of biodiversity [1–5]. These paradigmatic ideas share a common theme in that they were largely developed before adequate data were available to test them. That gap between theory and empiricism, however, began to narrow in the 1950s when nations poured massive resources into research and technology, paving the way for the transition from “little science to big science” [6]. Though primarily tailored for military and biomedical purposes, the advancements spilled over into other fields and opened up novel ways to tackle long-standing biological questions previously addressed only with mathematical models or restrictive experimental conditions. For instance, tech-savvy wildlife biologists began designing radio transmitters to track animals [7], and biochemically-inclined evolutionary biologists started developing tools to assay genetic variation in the wild [8,9]. These technological innovations, among countless others, revolutionized data collection in the biological sciences and had a lasting impact on our understanding of ecological and evolutionary processes.

Fast-forward to the present day, and these technologies have been replaced by smaller, faster, higher-throughput versions. Aided by the so-called “Information Technology revolution” (sensu [10]), the ability to collect and remotely access detailed data on wild organisms has changed the questions and scope of research in the biological sciences [11]. An estimated 50,000 wild animals are currently fitted with tracking devices [12], often sending real-time data directly to the researcher. Tracking technology is rapidly improving [13,14], with handling being minimized [15] and size reduced to the point where even invertebrates (as small as bumblebees) can be monitored remotely [16]. Analogously, evolutionary biologists now screen entire genomes of wild populations; for instance, in one recent study, Poelstra et al. [17] inferred evolutionary processes in natural crow populations on the basis of 1,700,000,000,000 base pairs of raw sequencing data. These novel datasets have already revealed insights into animal behavior [18,19], challenged evolutionary assumptions [20], and informed wildlife management and monitoring [21,22].

As major gains are being independently made in both fields (e.g., [13,23]), the parallel advancements of animal instrumentation and high-throughput sequencing hold great potential to reconcile animal behaviors and aspects of individual life history with ecological and evolutionary dynamics. Importantly, animal instrumentation data capture information on hitherto inaccessible phenotypic variability—often including the underlying physiological mechanisms—upon which natural selection can act (Table 1). The integration with high-throughput DNA sequencing data will elucidate the nature of the underlying genomic architecture of such traits and advance our understanding of fundamental ecological and evolutionary processes such as migration, foraging behavior, energetics, and communication in natural populations.

**Table 1:** Types of biological information that can currently be obtained from high-tech animal instrumentation (including automated image-based tracking).

Instrumentation	Biological information obtained from data
Tracking technology (e.g., GPS radio collars, light-based geolocators, passive acoustic arrays)	Migration patterns (timing, direction), habitat selection, energetic expenditure, temporal activity patterns, decision processes
Heart-rate monitors	Stress responses, physiological patterns
Accelerometers, time-depth recorders	Activity patterns, foraging or diving behavior
Acoustic recorders	Foraging behavior, communication, social interaction
Video recorders	Foraging behavior, habitat interface, social interaction
Contact collars	Social interaction
Temperature loggers	Daily and seasonal body temperature patterns, metabolism
Automated image-based tracking	Individual and species interactions, complex ecological patterns

Despite the wealth of phenotypic and behavioral data that can be generated from animal instrumentation, there exist only a handful of examples and tangential references as to how they can be analyzed in an evolutionary context or integrated with (population) genetic information. Merging these disparate datasets—including global remote sensing data at high spatial and temporal resolution [24]—produces a more holistic view on what structures populations and drives phenotypic variability in nature, and there is potential to develop new model systems and expand on existing biological theory [25,26]. In the following sections, we highlight the first attempts to integrate animal instrumentation with DNA sequence data (Table 2), provide a conceptual framework for integrating animal instrumentation and high-throughput sequencing, and list fundamental biological questions that might be addressed through this merger.

**Table 2:** Examples of questions addressed by integrating high-tech instrumentation with genetic data.

Basic biological question	Reference
How does the spatial ecological landscape influence gene flow?	[27,28]
What is the genetic basis of migratory behavior?	[29–31]
Does genetic structure reflect movement patterns or habitat use?	[32–34]
When is the use of habitat and resources a learned versus innate behavior?	[35]
Are animal social networks genetically structured?	[36–38]
How do social interactions and genetic relatedness impact disease transmission?	[39]

## Integration into Ecological Genetics

Ecological genetics is an integrative field of study focused on establishing a link between variation in environmental or phenotypic parameters and population genetic attributes (e.g., population differentiation, demographic history, adaptive genetic variation). Analyses can take various forms, such as landscape genetics, association studies, or comparative analyses: all essentially share the same premise of quantifying (or visualizing) the interaction between an ecological and genetic dataset (see Table 2). To date, the ecological component of these studies has typically consisted of a single location where animals were captured (with accompanying environmental parameters), only allowing for the assessment of broad-scale covariance between ecological or morphological parameters and genetic variation (e.g., [40]). As both instrumentation and genomic data can be obtained with increasing ease in a high number of individuals, the field of ecological genetics is expanding and moving from largely population-level summaries toward both ecosystem-wide and individual-based analyses.

**Fig 1:**
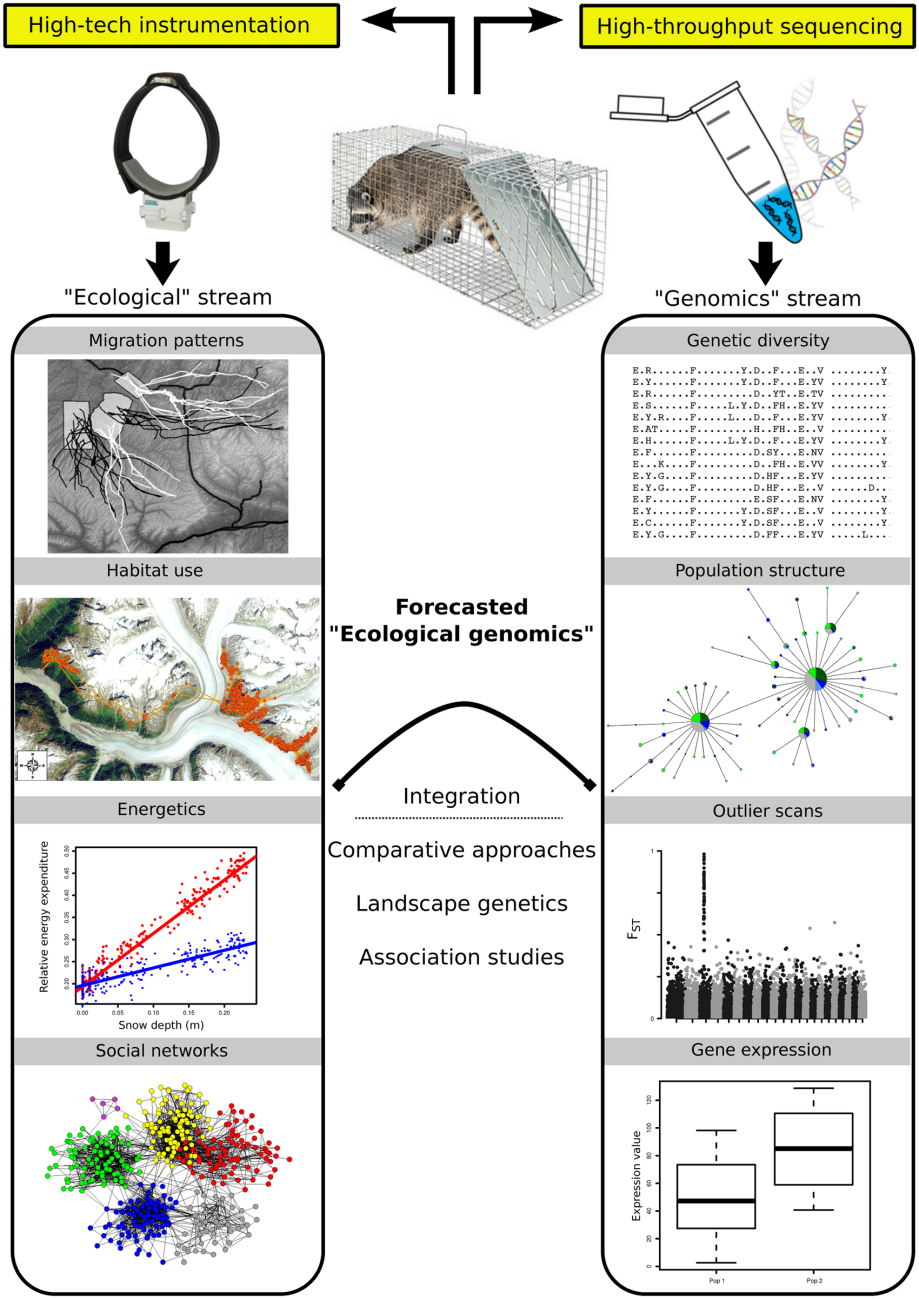
Conceptual overview of the integration of data from high-tech animal instrumentation with high-throughput sequencing data. The top section highlights that tissue collection for sequencing and instrumentation fitting naturally occur at the same time. The two side boxes show the separate analysis streams—ecological (left) and genomics (right). Integrative approaches making use of both data types are listed in the middle. Image contributors: raccoon in trap by Woodstream Corporation; map of habitat use was generated in ArcMap by Kevin White (Alaska Department of Fish & Game); radio-collar from LOTEK WIRELESS Inc.

To this end, we present a conceptual framework (Fig 1) that we view as a methodological partner to the more theoretical frameworks presented in Coulson et al. [41] and Ellegren and Sheldon [42]. The merger of instrumentation data with genomic data comes as a natural progression of traditional work in ecological genetics capitalizing on (i) mature methodology in two hitherto disparate research streams and (ii) the fact that both datasets are simultaneously obtained, in that it is common practice to sample DNA (blood, tissue, buccal swabs) when instrumenting an animal (see Fig 1). Below, we provide examples of key biological questions that will benefit from the integration of genomic data with various types of instrumentation data.

### Characterizing Species Interactions

The organization of natural communities is what drives ecosystem functions. Both environmental DNA (eDNA) and tracking technology provide compatible approaches to documenting species interactions. Image-based tracking visually captures encounters with other species and conspecifics [43] and can reveal predator–prey dynamics [44] along with fine-scaled resource selection or avoidance. High-throughput eDNA approaches can assay species communities, infer diet composition, and document invasive species [45]. In particular, contrasting eDNA catalogues with tracking-based assessments of species interactions and resource use will improve estimates of ecological niche breadth and overlap.

### Quantifying the Impact of Environmental Change

How changes in the environment have and will impact natural populations is the focus of much research and debate. Historical changes in a population’s effective size (*N*_e_) can be reconstructed by temporal sampling of DNA [46] or estimated from whole-genome sequence of a single individual [47]. Individual-based location data from animal instrumentation coupled with paleodistribution models is a powerful tool for inferring past ecological niches [48]. Integration of these separate streams has the potential to link changes in historical *N*_e_ with paleoecological niche reconstructions and, in turn, identify key climatic variables connected to past population changes (e.g., [49]). Newly developed community-level models relying on genomic and environmental variables (the latter based on instrumentation data and subsequent habitat models—see below) can then identify gene–environment relationships with the applied potential to earmark populations particularly vulnerable to environmental change [50].

### Understanding Animal Movement

Animals respond to their environment at different temporal and spatial scales. Dispersal (unidirectional movement between natal and breeding sites) and migration (cyclic, seasonal movement between breeding and non-breeding areas) are two key strategies animals employ in response to environmental and demographic stimuli. Instrumentation data can provide novel insight into migration routes [51] and the spatial partitioning and choice of habitat during migration [52], while genome scans have revealed candidate genes associated with migratory behavior [53]. Incorporating survival data further allows for assessing fitness differences across movement strategies and their population genetic consequences [54] or causes, predicated on the idea of fitness-associated dispersal [55]. Comparing runs of homozygosity (ROH; long stretches of invariant DNA sequence that are identical by descent) in dispersers and residents (non-dispersers)—identified either with genomic or tracking data—will allow for testing theoretical predictions on the role of inbreeding in the evolution of dispersal on a locus-by-locus basis [56]. Moreover, studies of collective (movement) behavior or social contagion (e.g., [57,58]) will benefit from an understanding of the genetic underpinnings of individual differences, such as boldness or leadership, in affecting movement dynamics.

### Gene Flow and Adaptive Divergence

Dispersal results in the exchange of individuals among breeding populations, and landscape genetics attempts to quantify how landscape variation influences this flow of genes [59]. Selecting and quantifying variables is challenging, subjective, and inherently biased by human perception. Tracking technology with coarse (geolocators) to fine-scale (GPS telemetry data) resolution fed into habitat selection models provides a more objective approach to scoring landscape features relevant to the organism [27,28]. Combined with environmental data from, for example, satellite imagery or habitat selection models, tracking and genomic data can identify patterns consistent with adaptive divergence [50,60]. Using instrument data as prior information to parameterize habitat features in a landscape genetics model will link often abstract population genetic parameters to real biological processes and guide hypotheses on local adaptation that form the basis of screening for adaptive genetic variation and studies of ecological speciation [40,61]. Furthermore, the combination of contemporary movement data and genomic estimates of admixture and gene flow will allow for testing fundamental ideas on the role of non-random dispersal on local adaptation and resource use [62].

### Altruism and Kin Selection

Theory predicts that population structuring is a prerequisite for altruistic behavior to evolve. Kin selection theory puts a premium on genetic relatedness [63], and game theoretical approaches model the evolution of altruism on structured social interaction networks [64]. While the relationship between social structure, genetic relatedness, and their evolutionary consequence has been extensively treated in the theoretical literature, empirical contributions remain scare [65,66]. Data from proximity loggers, GPS tracking, and automated image systems allow for social interactions to be quantified remotely [36,38]; large-scale genomic data allows accurate estimates of genetic relatedness [67]. The combination of behavioral data with individual whole-genome sequences also has the exciting prospect of unveiling the actual loci underlying altruistic behavior (e.g., green-beard genes [68]).

### Mechanisms of Pathogen Transmission

The relative risk of pathogen transmission in wild populations is often inferred with population genetic models [69] or, alternatively, by attempting to link animal contact rates and relatedness to transmission probabilities [39] or pathogen population structure [70]. Similar to the estimation of population connectivity mentioned above, the combination of genomic and instrument data should improve predictive power if integrated into a modeling framework that screens host and pathogen genomes. Furthermore, researchers studying humans have recognized the clinical relevance of detecting ROH [71], and domestic animal researchers have found links to disease in case and control studies [72]. In the wildlife disease context, it is conceivable that the frequency of social interactions, use of point resources, or general range overlap (all inferred from instrumentation) might be, in part, mediated by specific ROH or genomic regions.

### Genotype:Phenotype Correlations

Charting the genetic basis of phenotypic variation relevant to fitness is key to furthering our understanding of ecological and evolutionary processes in the wild [42]. Screening phenotypes derived from instrumentation data goes beyond standard biometric or coloration traits that are often the focus in these studies, and genome data gives the individual-based resolution required to uncover the genomic architecture of such traits. Virtually all phenotypes obtainable through instrumentation data—ranging from vigilance behavior to hibernation period—might have substantial narrow-sense heritability (i.e., phenotypic variation explained by specific alleles). Migratory behavior is a prime example in which substituting population-based approaches (of linking allele frequencies with phenotypic proxies by stable isotope biomarkers) with individual-based instrumentation and genomic data is expected to make a difference [73,74]. We should point out that underlying genomic architecture dictates the power of such scans [75], and there are cases (e.g., polygenic traits) in which genotype:phenotype signals will be virtually impossible to disentangle from noise without a large sample size. Any association will likely require functional studies to have biological significance beyond detecting a candidate genetic basis.

### A Role for Gene Expression

Identical protein sequences can have different phenotype effects, depending on their relative abundances [76]. Linking mRNA gene expression patterns (dictating protein abundance to a large degree) to phenotypic differences (e.g., activity budgets, response to stressors) will aid in characterizing trait plasticity and prescreening potential targets of selection. For now, this approach is largely restricted to common garden approaches, but could enter natural settings under selective or innovative sampling regimes. Similarly, new sequencing technology allowing the characterization of epigenetic inheritance patterns provides another exciting opportunity to link differences in gene regulation to phenotypic variability as displayed in the wild.

## Challenges for Implementation

Despite the apparent synergies between animal instrumentation and sequencing data, there are reasons why this integration has been hampered. Primarily, both are young types of data, with their respective fields still struggling with data management and streamlined analytical pipelines. Below, we expand on the primary roadblocks and reflect on possible solutions as we see them.

### Knowledge and Collaboration

The training, background, and expertise needed to analyze these disparate datasets are unlikely to exist in a single lab or research group. In Shafer et al. [27], two very different datasets and analyses were combined: a population genetic study and an analysis of habitat selection. These studies can be demarcated as “genetic” and “ecological,” and historically would have been published separately (i.e., the streams in Fig 1). Understanding the nuances of habitat selection and population genetic theories require separate schools of training, and their combination is an undertaking that cannot be achieved without collaboration. As many instrumentation studies already require expertise from engineering and physics, and genomics similarly requires diverse expertise, from specialized lab technicians to bioinformaticians, the need for multidisciplinary collaborations is at a premium.

### Informatics Issues and the Data Deluge

The amount of data being generated by both of these data streams is a major challenge. Storing, analyzing, and archiving genomic data is already a hurdle, requiring massive amounts of storage and CPU hours that are generally only available on high-performance computing resources (see [77]). There are also challenges associated with analyzing and understanding these new datasets that were simply not present with more “traditional” ecological and genetic datasets [78,79] and that will only become more profound with their integration. In many instances, the challenges have been recognized and are active areas of research and debate, simply requiring time to be resolved. A critical step will be to link existing databases (such as Movebank and GenBank) to allow researchers easy access to all biological data available on their focal species.

### Financial Considerations

Re-sequencing individual genomes is still costly, although prices have rapidly declined [80]. If the focal species does not have a reference genome (which will limit the available analyses), researchers might choose to assemble a draft genome, a task that is both expensive and requires significant expertise [81]. The logistical and financial requirements to capture and fit an animal with a GPS radio collar and camera can also be substantial. Multiply these costs by twenty to achieve a modest sample size, and we have easily exceeded most research group budgets for the foreseeable future. There are cheaper alternatives, for example, geolocators and reduced representation genome sequencing, but they come at the expense of resolution and, thus, might not be appropriate for addressing some of the aforementioned questions. Many wildlife agencies regularly capture and instrument animals; thus, academic–agency collaborations provide a key opportunity to navigate this financial obstacle.

## Conclusion

Technological innovations take time to trickle down to basic biological research, but we are now in the midst of a data revolution stemming from recent high-tech and throughput advancements. There are several promising fundamental research questions that are tenable from the merger described in this essay, and there is clear potential to link largely disparate schools of thought. Twenty years ago, an essay about sequencing genomes and remotely tracking animals across the globe in real time would have been the subject of science fiction. In 2015, there are over 50,000 animals being tracked [12], and single research groups now sequence dozens, up to hundreds, of individual genomes [17,82]. By embracing new technology and integrating these data streams into an ecological genomic framework (Fig 1), we are now poised to inform, challenge, and develop biological theory.
